# Utility of specific bioelectrical impedance vector analysis for the assessment of body composition in children

**DOI:** 10.1016/j.clnu.2020.07.022

**Published:** 2021-03

**Authors:** Jonathan CK. Wells, Jane E. Williams, Leigh C. Ward, Mary S. Fewtrell

**Affiliations:** a Childhood Nutrition Research Centre, Population, Policy and Practice Reseach and Teaching Department, UCL Great Ormond Street Institute of Child Health, 30 Guilford Street, London, WC1N 1EH, UK; b School of Chemistry and Molecular Biosciences, The University of Queensland, Brisbane, Australia

**Keywords:** Body composition, Bioelectrical impedance analysis, Children, Adolescents, Bioelectrical impedance vector analysis

## Abstract

**Background *&* aims:**

Bioelectrical impedance analysis (BIA) is widely considered a body composition technique suitable for routine application. However, its utility in sick or malnourished children is complicated by variability in hydration. A BIA variant termed vector analysis (BIVA) aims to resolve this, by differentiating hydration from cell mass. However, the model was only partially supported by children's data. To improve accuracy, further adjustment for body shape variability has been proposed, known as specific BIVA (BIVA_specific_).

**Methods:**

We re-analysed body composition data from 281 children and adolescents (46% male) aged 4–20 years of European ancestry. Measurements included anthropometry, conventional BIA, BIVA outcomes adjusted either for height (BIVA_conventional_), or for height and body cross-sectional area (BIVA_specific_), and fat-free mass (FFM) and fat mass (FM) by the criterion 4-component model. Graphic analysis and regression analysis were used to evaluate different BIA models for predicting FFM and FM.

**Results:**

Age was strongly correlated with BIVA_conventional_ parameters, but weakly with BIVA_specific_ parameters. FFM correlated more strongly with BIVA_conventional_ than with BIVA_specific_ parameters, whereas the opposite pattern was found for FM. In multiple regression analyses, the best prediction models combined conventional BIA with BIVA_specific_ parameters, explaining 97.0% and 89.8% of the variance in FFM and FM respectively. These models could be further improved by incorporating body weight.

**Conclusions:**

The prediction of body composition can be improved by combining two different theoretical models, each of which appears to provide different information about the two components FFM and FM. Further work should test the utility of this approach in pediatric patients.

## 1 Introduction

There is increasing interest in the assessment of body composition in children, for several reasons. Body composition measurements could inform clinical diagnosis, improve routine management, help determine nutritional and fluid requirements as well as some therapeutic doses, and assess longer-term cardio-metabolic risk [1]. However, obtaining accurate measurements is challenging in sick or malnourished children, who often cannot comply with demanding protocols. For many decades, routine body composition assessment was restricted to simple anthropometry, such as body mass index (BMI), skinfold thicknesses and body circumferences. Recently, more sophisticated methods have become available, including air-displacement plethysmography, dual-energy X-ray absorptiometry and isotope dilution [2,3], but remain restricted to specialist research centres. There is still a need for simpler methods that can be widely used, especially in community studies [2]. In this context, bioelectrical impedance analysis (BIA) has long attracted interest. The method involves passing a very small imperceptible current between different parts of the body and measuring the resistance of the tissues. Raw data are quick and easy to collect, though individuals must stay still in standardised position and relaxed state for a few seconds.

Conventionally, whole-body impedance (Z) is measured between the wrist and the ankle. The height (H)–adjusted impedance index (H^2^/Z) is then a strong predictor of either total body water (TBW) or fat-free mass (FFM) [2]. Resistance is more often measured in place of Z; though similar in magnitude, it is more closely related to TBW (see below). However, associations of H^2^/Z (or R) with TBW or FFM vary between populations, and population-specific calibration studies are recommended. Even then, subtle variation in traits such as body proportions (e.g., the ratio of limb to trunk length), maturation state and ethnic ancestry result in significant random error in individuals [[4], [5], [6], [7], [8]]. Since FM is typically the smaller component of weight, random error on FFM is relatively greater when propagated to FM. Several solutions to this predicament have been suggested, such as incorporating more predictors in calibration equations, conducting segmental analysis or using different bioelectrical frequencies to predict different fluid compartments, but none of these approaches has substantially reduced predictive error [[9], [10], [11]].

An alternative approach, developed by Piccoli et al. and known as bioelectrical impedance vector analysis (BIVA), divides Z into its components, resistance (R) and reactance (Xc), normalizing each for H [12]. Based on bioelectrical theory, R is expected to correlate negatively with body fluids, whereas Xc should correlate positively with body cell mass [13]. On this basis, if the data are plotted on ‘R/H-Xc/H’ graphs, data from a population will form an ellipse as expected for bivariate data, where one diagonal axis represents variability in hydration, and the orthogonal axis variability in body cell mass, closely correlated with FFM [13]. A key advantage of Piccoli's approach is that no predictive equations are required, however the resulting data are both qualitative and semi-quantitative, and still require some form of processing to aid interpretation. For example, BIVA traits vary with age, which may in part by due to changes in body size [14]. As one solution to this, we previously published BIVA reference data for children and adolescents aged 5–20 years, allowing the use of age- and sex-standardized z-scores [15].

Recently, studies of disease states have supported several assumptions of BIVA theory [16,17], however in healthy children the findings were only partially supportive [15]. While BIVA variables correlated as expected with hydration, they did not correlate with FFM. A new variant known as specific BIVA (BIVA_specific_), aims to improve the correlation with body composition by adjusting R and Xc not only for height, but also for body cross-sectional area [18,19]. This addresses fundamental bioelectrical theory, since according to Ohm's law, the resistance of a conductor (e.g., a cylinder) to a current varies proportionally with its length but inversely with its cross-sectional area [18,19]. Research in adults has shown that BIVA_specific_ measurements correlate strongly with % fat as opposed to FFM [18].

We therefore aimed to evaluate specific BIVA data in a large dataset of body composition in children from a wide range of nutritional status, which we previously analysed for our assessment of conventional BIVA. We also conducted exploratory analyses, combining both conventional BIA and specific BIVA approaches to see if they contributed independently to the prediction of body composition.

## 2 Methods

The data has been described in detail previously [15], and came from two prior studies conducted by our group, both approved by the Ethical Committee of UCL Institute of Child Health and Great Ormond Street Hospital. Informed consent was obtained from all participants and/or their parents as appropriate. For this analysis, we included children of European ancestry only, as ethnicity has been associated with variability in fat and lean distribution [6,20] and the loci of BIVA ellipses [21].

### 2.1 Participants

Most individuals were from a study of healthy children/adolescents aged 4–20 years, with no BMI exclusion criteria except that they could not be attending a weight loss clinic, and they must not have any disease that might affect growth and development. Baseline data from obese children aged 7–14 years participating in weight loss intervention studies were also used. In combination, the two datasets cover a wide range of BMI [22,23].

### 2.2 Data collection

Anthropometry and body composition were measured as described previously, with FFM and FM calculated using the 4-component model [24]. Single-frequency BIA was conducted at 50 kHz (Quadscan 4000 instrumentation; Bodystat, UK). This frequency is proposed to maximise signal-to-noise ratio and minimise frequency-dependent errors and variability of electric flow paths [25,26], though the optimal frequency also varies between individuals and by age [27]. Participants lay supine on a non-conducting couch. Disposable EKG-style Ag/AgCl gel electrodes were attached in standard tetrapolar manner to left hand and foot [28]. Z, R, Xc and PA were recorded in duplicate, and the average used in analyses. The device was regularly calibrated, and on all occasions the device was within the manufacturer's specifications.

As usual in the conventional BIVA (BIVA_conventional_) approach, R and Xc were standardised for height (H) and expressed as R/H and Xc/H in ohm (Ω)/m [12]. Prior to analysis, we excluded individuals with PA > 8.0 (values in healthy people range between 5° and 7°, hence allowing for measurement error, values above 8° were considered implausible; n = 14 excluded) [29], as well as those with poor repeatability (exclusion criteria were duplicates >0.5 for PA, and ≥6.0 for R/H and Xc/H; n = 25 excluded). In those data retained for analysis, technical error of the mean calculated using the formula of Ulijaszek and Kerr [30] was 1.9 Ω for Z and R and 1.1 Ω for Xc.

The new data incorporated in this analysis were body girths, which were available for all but one of the subjects in the previous analysis. Girths were measured for the mid-upper arm, waist and maximal calf, using a non-stretchable tape. Technical error of the mean for girth data in our research centre is 0.2 cm for waist and 0.1 cm for arm and calf girth. The raw data are available as Supplementary online Data.

### 2.3 Data processing and statistical analysis

Three age groupings were derived, broadly representing pre-pubertal (4–9 years), pubertal (10–14 years) and post-pubertal (15–20 years) individuals. Assessment of nutritional status was based on UK BMI z-scores, using cut-offs of <−2 to define thinness, >1 to define overweight and >2 to define obesity.

For conventional BIA, the impedance index was calculated as the square of height divided by Z (HT^2^/Z) in cm^2^/ohms. For specific BIVA (BIVA_specific_), following the approach of Buffa and colleagues [18], cross-sectional areas (A) of the arm, torso and leg were first calculated as follows:

Equation 1: A = [girthˆ2]/(4∗pi)

These were then summed, again as recommended [18], to give a whole body area correction factor as follows:

Equation 2: Area [A] = (0.45 ∗ arm area) + (0.1 ∗ waist area) + (0.45 ∗ leg area)

We obtained a value for length from height, again as recommended [18]:

Equation 3: Length [L] = height (m) ∗ 1.1

In BIVA_specific_, R and Xc are then multiplied by the correction factor, A/L, to give R_specific_ and Xc_specific_ respectively.

Graphs were plotted with sex-specific LOESS lines, fitted with 75% span, for BIVA_conventional_ and BIVA_specific_ outcomes against age, and for FFM and fat mass against BIVA_conventional_ and BIVA_specific_ parameters. Correlations between conventional or specific BIVA parameters and body composition outcomes were calculated. Multiple regression models were used to test independent associations of conventional BIA and BIVA_conventional_ and BIVA_specific_ parameters with body composition outcomes, adjusting for age and sex (males coded 1, females coded 2).

We developed a series of multiple regression models, intended to reveal the differing associations of anthropometry and different BIA approaches with the two body composition outcomes. We evaluated the different models in terms of the proportion of variance in the outcome explained (adjusted r^2^ value, which aids comparison across models) and the standard error of the estimate (SEE) in individuals. To aid comparisons across models, we also provide the t-statistic for each individual predictor, and the overall F-statistic of each model.

Baseline models included only age and sex, in order to help understand how the addition of any further anthropometric or BIA parameters improved the accuracy of predicting outcomes. We then developed models that introduced only anthropometric parameters (either weight, or the three girths). Subsequent models introduced either conventional BIA parameters (HT^2^/Z), or conventional BIVA parameters (R/H, Xc/H), or specific BIVA parameters (R_specific_, Xc_specific_). Finally, we developed ‘combined models’ incorporating both conventional BIA and specific BIVA parameters, as well as testing the addition of weight. All graphs and analyses were run in IBM SPSS Statistics, version 24.

## 3 Results

After data cleaning, full data were available for 281 individuals, comprising 130 boys and 151 girls. The average age was 11.8 (SD 3.7) years, range 4.2–19.9 years. There was no difference in average age between the sexes. Table 1 presents values for raw anthropometry, body composition and BIA values stratified by age group and sex. There were sex differences in body size, shape and composition, especially in younger and older children, which may reflect the greater representation of obesity in the middle age group. Most traits varied significantly by age. Regarding nutrition status, BMI z-score ranged from −3.3 to +4.3, and the sample included 9 (3.2%) who were underweight, 161 (57.3%) who were normal weight, 49 (17.4%) who were overweight, and 62 (22.1%) who had obesity.

**Table 1:** Anthropometry, body composition and bioelectrical variables stratified by age and sex.

Males	4–9 years (n = 31)		10–14 years (n = 73)		15–20 years (n = 26)		*p*-value^b^
Trait	Mean	SD	Mean	SD	Mean	SD	
Height (cm)	122.3^a^	11.9	154.1	11.4	178.8^a^	6.5	<0.0001
BMI (kg/m^2^)	17.4^a^	3.8	20.7^a^	5.3	23.8	8.7	<0.0001
R50	683.7	64.6	604.1	85.4	514.0^a^	75.0	<0.0001
Xc50	62.9	7.4	61.0	8.9	60.8^a^	8.2	0.55
Arm girth (cm)	19.5^a^	3.5	25.1^a^	5.0	29.5	7.1	<0.0001
Waist girth (cm)	59.2	9.6	72.2	14.3	82.3	20.4	<0.0001
Calf girth (cm)	26.0^a^	3.4	32.5	4.4	37.0	4.5	<0.0001
Fat-free mass (kg)	20.3	4.5	36.3	9.1	58.9^a^	8.4	<0.0001
Fat mass (kg)	6.4^a^	6.5	13.8^a^	11.5	17.2	20.6	0.005
Females	4–9 years (n = 59)		10–14 years (n = 67)		15–20 years (n = 25)		
Trait	Mean	SD	Mean	SD	Mean	SD	*p*-value^b^
Height (cm)	129.2	10.4	155.4	9.3	165.3	7.6	<0.0001
BMI (kg/m^2^)	19.4	5.2	23.0	6.9	24.8	7.2	<0.0001
R50	686.7	85.5	616.7	83.6	612.6	71.2	<0.0001
Xc50	65.9	7.9	61.8	7.4	65.8	9.2	0.009
Arm girth (cm)	22.6	4.9	27.1	5.7	29.4	5.5	<0.0001
Waist girth (cm)	63.5	12.7	74.2	15.6	78.2	15.6	<0.0001
Calf girth (cm)	28.3	4.7	33.8	5.4	36.8	4.2	<0.0001
Fat-free mass (kg)	22.7	5.6	36.6	7.7	43.7	6.0	<0.0001
Fat mass (kg)	10.9	8.3	19.4	13.3	24.2	14.3	<0.0001

a Difference between the sexes significant p < 0.05, by independent samples t-test.

b Difference between the age groups, tested by ANOVA.

**Fig. 1:**
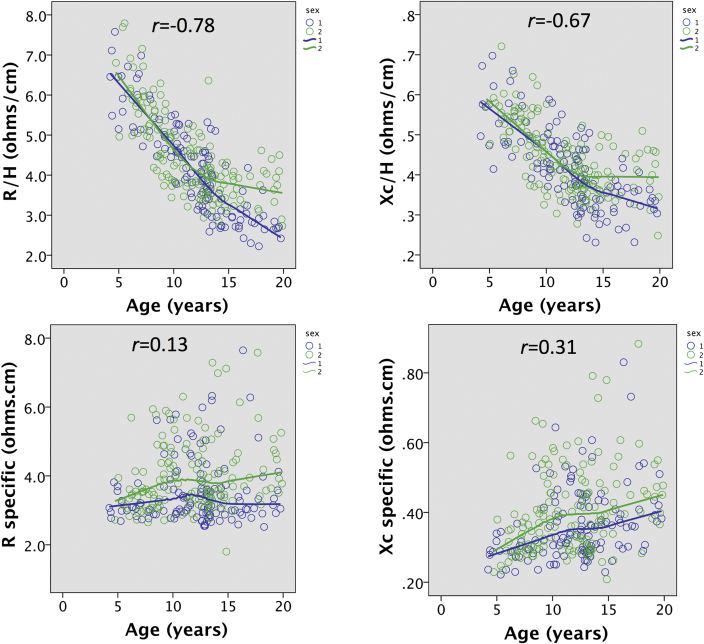
Associations of BIVA_conventional_ parameters (R/H and Xc/H) and BIVA_specific_ parameters (R-specific, Xc-specific) with age, stratified by sex.

Figure 1 illustrates associations of BIVA_conventional_ and BIVA_specific_ parameters with age. Adjusted for height, BIVA_conventional_ parameters were strongly associated with age (R/H *r* = −0.78; Xc/H *r* = −0.67; both p < 0.0001), whereas BIVA_specific_ parameters were more weakly associated (R_specific_ *r* = 0.13, p = 0.027; Xc_specific_ *r* = 0.31, p < 0.0001). For BIVA_conventional_ parameters, the sexes showed very similar associations by sex until around 12 years, when values stopped declining with age in females but continued to decline in males. Conversely, BIVA_specific_ parameters were consistently higher in females compared to males at all ages, with this difference increasing with age for R_specific_.

**Fig. 2:**
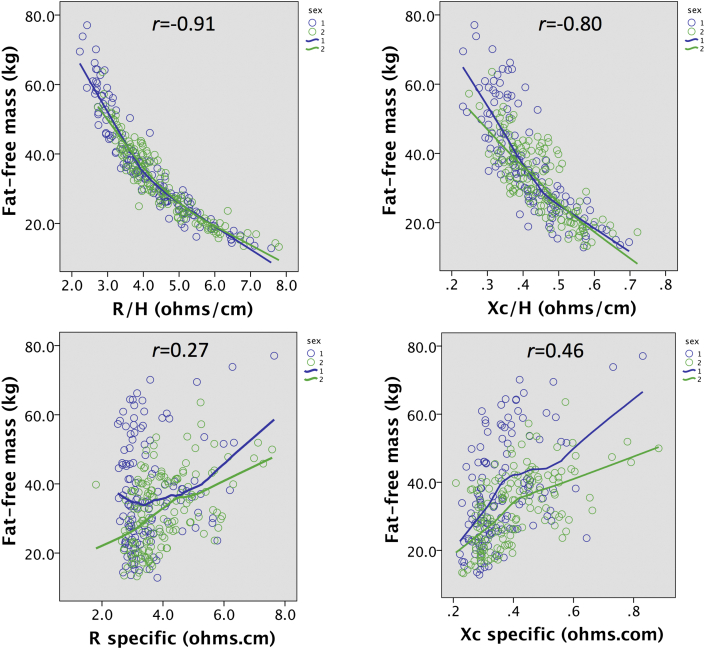
Associations of BIVA_conventional_ parameters (R/H and Xc/H) and BIVA_specific_ parameters (R-specific, Xc-specific) with absolute fat-free mass (FFM) measured by the 4-component model, stratified by sex.

Figure 2 illustrates correlations of BIVA_conventional_ and BIVA_specific_ parameters with absolute FFM. For BIVA_conventional_ parameters, there were strong and relatively tight negative associations in both sexes, that were slightly curvilinear (R/H *r* = −0.91; Xc/H *r* = −0.80; both p < 0.0001). In contrast, BIVA_specific_ parameters were positively correlated with FFM, though relatively weakly (R_specific_ *r* = 0.27; Xc_specific_ *r* = 0.46; both p < 0.0001), and here the male FFM values tended to be greater than those of females for a given BIVA value, though this difference was not linear. Overall, BIVA_conventional_ parameters correlated with FFM substantially better than did specific BIVA_specific_ parameters.

**Fig. 3:**
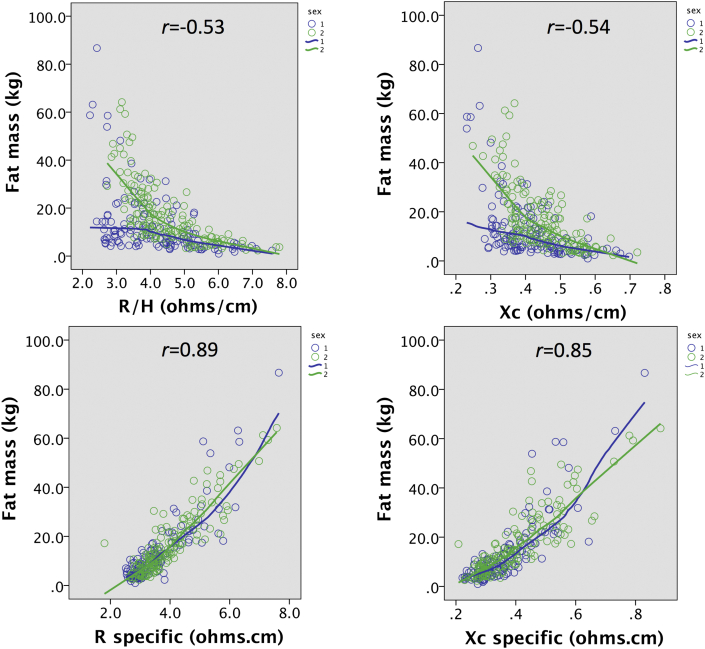
Associations of BIVA_conventional_ parameters (R/H and Xc/H) and BIVA_specific_ parameters (R-specific, Xc-specific) with absolute fat mass (FM) measured by the 4-component model, stratified by sex.

Figure 3 illustrates equivalent correlations of BIVA_conventional_ and BIVA_specific_ parameters with absolute FM, where the contrast between the two analytical approaches took the opposite pattern. For BIVA_conventional_ parameters, there were moderately strong negative associations in both sexes, that were linear in males but curvilinear in females (R/H *r* = −0.53; Xc/H *r* = −0.54; both p < 0.0001). In contrast, BIVA_specific_ parameters were positively and tightly correlated with FM, with the association almost linear for R_specific_ and weakly curvilinear for Xc_specific_ (R_specific_ *r* = 0.89; Xc_specific_ *r* = 0.85; both p < 0.0001). Here the sex differences were negligible. Overall, BIVA_specific_ parameters correlated with FM substantially better than did BIVA_conventional_ parameters.

Table 2 presents multivariable regression models for associations of BIVA_conventional_ and BIVA_specific_ parameters with body composition outcomes, taking into account age, sex and weight. To compare the additional information provided by the two different BIVA approaches, the first model included only age and sex. This model explained 72.2% of the variance in FFM, with an SEE of 6.87 kg, and 15.8% of the variance in FM, with an SEE of 12.13 kg. The model had overall F-values of 365 and 27 for FFM and FM respectively.

**Table 2:** Prediction of Fat-free mass and fat mass from age, sex and conventional BIA and BIVA and/or specific BIVA parameters.

	Fat-free mass (kg)							Fat mass (kg)						
	Beta	SE	*t*	*p*	*r*^2^	F	SEE (kg)	Beta	SE	*t*	*p*	*r*^2^	F	SEE (kg*)*
**No BIA**														
Constant	5.097	1.920	2.65	0.008	0.722	365	6.87	−8.038	3.389	−2.37	0.018	0.158	27	12.13
Age (years)	2.908	0.110	26.42	<0.0001				1.323	0.194	6.81	<0.0001			
Female sex	−3.288	0.824	−3.99	<0.0001				4.763	1.454	3.275	0.001			
Constant	6.052	1.187	5.10	<0.0001	0.894	788	4.25	−6.031	1.187	−5.08	<0.0001	0.897	813	4.24
Age (years)	1.543	0.094	16.49	<0.0001				−1.545	0.094	−16.52	<0.0001			
Female sex	−3.765	0.510	−7.39	<0.0001				3.760	0.509	7.383	<0.0001			
Weight (kg)	0.322	0.015	21.23	<0.0001				0.678	0.015	44.65	<0.0001			
Constant	−12.657	2.282	−5.55	<0.0001	0.850	319	5.04	−42.641	1.746	−24.42	<0.0001	0.915	601	3.86
Age (years)	1.926	0.112	17.25	<0.0001				−0.296	0.085	−3.46	0.001			
Female sex	−4.299	0.659	−6.53	<0.0001				3.732	0.504	7.40	<0.0001			
Arm girth (cm)	0.299	0.724	1.34	0.084				0.529	0.171	3.09	0.002			
Waist girth (cm)	0.016	0.071	0.23	<0.0001				0.663	0.055	12.16	<0.0001			
Calf girth (cm)	0.695	0.155	4.48	0.004				−0.156	0.119	−1.32	0.18			
**Conventional BIA**														
Constant	−1.845	0.707	−2.61	0.010	0.964	2523	2.46	−14.191	3.079	−4.61	<0.0001	0.341	49	10.73
Age (years)	0.430	0.069	6.21	<0.0001				−0.873	0.302	−2.89	0.004			
Female sex	0.260	0.306	0.85	0.3				7.908	1.335	5.92	<0.0001			
Height^2^/Z	0.820	0.019	43.43	<0.0001				0.727	0.082	8.84	<0.0001			
**Conventional BIVA**														
Constant	54.371	3.095	17.57	<0.0001	0.870	470	4.70	51.870	6.926	7.49	<0.0001	0.367	42	10.51
Age (years)	1.232	0.121	10.20	<0.0001				−0.520	0.270	−1.93	0.055			
Female sex	−1.658	0.571	−2.90	0.004				6.726	1.277	5.27	<0.0001			
R/H (ohms/cm)	−6.706	0.602	−11.13	<0.0001				−4.187	1.348	−3.11	0.002			
Xc/H (ohms/cm)	−6.64	6.184	−1.07	0.28				−53.184	13.837	−3.84	<0.0001			
**Specific BIVA**														
Constant	1.322	2.033	0.65	0.5	0.781	251	6.10	−37.218	1.718	−21.67	<0.0001	0.848	391	5.16
Age (years)	2.529	0.111	22.77	<0.0001				0.867	0.094	9.23	<0.0001			
Female sex	−4.333	0.751	−5.77	<0.0001				−0.285	0.634	−0.449	0.6			
R_specific_ (ohms.cm)	−2.121	0.927	−2.29	0.023				11.037	0.784	14.08	<0.0001			
Xc_specific_ (ohms.cm)	46.742	8.494	5.50	<0.0001				2.052	7.177	0.286	0.7			
**Combined model 1**														
Constant	−3.035	0.762	−3.98	<0.0001	0.970	1800	2.27	−39.501	1.420	−27.81	<0.0001	0.898	493	4.22
Age (years)	0.460	0.065	7.12	<0.0001				−0.217	0.120	−1.80	0.073			
Female sex	−0.327	0.295	−1.11	0.26				1.814	0.550	3.30	0.001			
Height^2^/Z	0.773	0.019	41.55	<0.0001				0.405	0.035	11.68	<0.0001			
R_specific_ (ohms.cm)	−0.737	0.347	−1.07	0.28				11.953	0.647	18.46	<0.0001			
Xc_specific_ (ohms.cm)	12.874	3.258	3.95	<0.0001				−15.694	6.072	−2.585	0.010			
**Combined model 2**														
Constant	5.669	1.336	4.24	<0.0001	0.975	1821	2.06	−5.641	1.336	−4.22	<0.0001	0.976	1870	2.06
Age (years)	0.410	0.059	6.93	<0.0001				−0.413	0.059	−6.98	<0.0001			
Female sex	−0.631	0.271	−2.33	0.021				0.630	0.272	2.32	0.021			
Height^2^/Z	0.532	0.036	14.85	<0.0001				−0.532	0.036	−14.83	<0.0001			
R_specific_ (ohms.cm)	−2.742	0.443	−6.19	<0.0001				2.737	0.443	6.18	<0.0001			
Xc_specific_ (ohms.cm)	13.454	2.965	4.54	<0.0001				−13.439	2.967	−4.53	<0.0001			
Weight (kg)	0.205	0.027	7.62	<0.0001				0.796	0.027	29.64	<0.0001			

Z – impedance, R – resistance, Xc – reactance, H - height.

R_specific_ and X_specific_ – R and Xc adjusted for both height and cross-sectional area.

SE – standard error, SEE – standard error of the estimate.

Adding in weight to this model improved accuracy of the prediction for both outcomes, producing identical r^2^ (89.4%) and SEE values (4.25 kg). This model had overall F-values of 788 and 813 for FFM and FM respectively. Adding in the three girths instead of weight further improved accuracy for predicting FM (r^2^ 91.5%, SEE 3.86 kg) but reduced the accuracy of predicting FFM (r^2^ 85.0%, SEE 5.04 kg). The model had overall F-values of 319 and 601 for FFM and FM respectively. This model indicates that upper body girths primarily predict fatness, with waist being the strongest predictor followed by arm, whereas calf was not significant. Instead, calf girth was the only girth that was a significant predictor of FFM.

Removing weight, the next model represented a conventional approach to BIA, and therefore included HT^2^/Z alongside age and sex. This model explained 89.4% of the variance in FFM, with an SEE of 2.46 kg, and 34.1% of the variance in FM, with an SEE of 10.73 kg. The model had overall F-values of 2523 and 49 for FFM and FM respectively.

Dropping HT^2^/Z, and adding instead the two BIVA_conventional_ parameters to the model, only R/H contributed significantly for FFM whereas both terms contributed for FM. The proportion of variance explained was lower for FFM (87.0%) but higher for FM (36.7%), while the SEE value increased substantially for FFM (4.70) and decreased slightly for FM (10.51 kg). The model had overall F-values of 470 and 42 for FFM and FM respectively.

Replacing BIVA_conventional_ with BIVA_specific_ parameters, the proportion of variance for FFM decreased further (78.1%), while the SEE increased substantially (6.10 kg). However the prediction was greatly improved for FM (r^2^ 84.7% and SEE 5.16 kg). The model had overall F-values of 251 and 391 for FFM and FM respectively.

The next model combined both BIVA_conventional_ and BIVA_specific_ terms. For FFM, the contributions were broadly additive, though R_specific_ did not contribute significantly to the model. The model explained 97.0% of the variance in FFM, with an SEE of 2.27 kg. For FM, both R_specific_ and Xc_specific_ contributed to the model, which explained 89.8% of the variance with an SEE of 4.22 kg. The model had overall F-values of 1800 and 493 for FFM and FM respectively.

Finally, weight was added back to the model, which resulted in the r^2^ and SEE being identical for FFM and FM models. The coefficients of the predictor variables were also near-identical but with opposite signs, the exception being weight which showed a higher coefficient, suggesting it plays a more important role in predicting FM than FFM. All terms were significant in both models, which explained 97.5% of the variance in FFM and FM with an SEE of 2.06 kg. The model had overall F-values of 1821 and 1870 for FFM and FM respectively.

The F-values for both the combined models are slightly lower than that for FFM in the conventional BIA model (F = 2523), but this reflects the inclusion of more predictors. In terms of the proportion of variance explained and SEE, the two combined models provided the most accurate predictions of FFM, and the second combined model provided the most accurate prediction of FM.

## 4 Discussion

The conventional approach to predicting body composition from BIA relies on the close association of height-adjusted resistance or impedance with body components that conduct electricity, the most obvious of which are TBW or FFM. These associations are strong in any given population, though the slope varies between populations according to age, maturation state and ethnicity [6,8]. Using carefully designed calibration studies, where children are sampled in equal numbers across a wide range of nutritional status, the SEE in individuals can be reduced to 1 kg of FFM [31], meaning that predicted values lie within ±2 kg of the ‘true’ value. In our study, the best SEE value using conventional BIA was slightly greater (2.46 kg of FFM), but this is due in part to our including a very wide range of both age and BMI, and we expect that better accuracy could be obtained using our approach in more homogeneous populations.

Our aim was to see if we could improve the prediction of FFM, using approaches based on BIVA. Expressing BIA data as height-adjusted vectors, using the BIVA_conventional_ approach, did not show strong associations with FFM, while for FM the approach was no better than that using conventional BIA. Indeed, no simple BIA model performed better for FM than a model containing only age, sex and three body girths. It has previously been recognised that BIVA_conventional_ parameters remain strongly associated with body shape, as impedance of body components is influenced by both cylinder length and cross-sectional area [18,19]. We therefore tested a new variant of BIA [18,19], which adjusts impedance for body cross-sectional area as well as length, with the aim of addressing more effectively variability in body morphology.

We first showed that two girths (arm and waist) were significant predictors of FM but not FFM, whereas the reverse scenario was apparent for calf girth. This supports the notion that incorporating upper body girths into regression models addresses variability in fatness. This is similar to data from adults, where for example girths tend to show higher correlations with FM than skeletal muscle mass, especially in females, though the arm is an exception to this pattern [32]. Consistent with that, we found overall that BIVA_specific_ parameters were more accurate predictors of FM than BIVA_conventional_ parameters, though this pattern was reversed for FFM. Nonetheless, even though the SEE for FM was substantially reduced, it was still large (5.16 kg), and the equivalent SEE for FFM (6.10 kg) compared very poorly compared to that obtained by conventional BIA. Thus, incorporating girths into conventional BIVA models improves the sensitivity of BIVA variability to body fat content, but without improving the accuracy of FFM prediction.

Intriguingly, the prediction models showed improvement when the conventional BIA and BIVA_specific_ parameters were combined. This approach resulted in the lowest SEE values for both FFM and FM (2.27 kg and 4.22 kg respectively) compared to either conventional BIA, BIVA_conventional_ or BIVA_specific_, while the r^2^ values were also the highest across these models (97.0% and 89.8% respectively). The BIA parameters appeared broadly additive in these models, indicating that the two theoretical approaches extract different information from the raw bioelectrical data, and that each component of information improves the prediction of the two main components of body composition.

Adding weight to this model generates identical r^2^ and SEE values, because of it summing FFM and FM. In each case, the r^2^ value is 97.5% and the SEE is 2.06 kg. This approach may be particularly successful in our own sample due to the high range of BMI, and further work is needed to test the effect of including weight in other samples that have lower degrees of BMI variability.

Our study shows for the first time in healthy children covering a wide range of nutritional status that BIVA_specific_ performs more poorly than conventional BIA in predicting FFM, but performs substantially better in predicting FM. This suggests that correcting bioelectrical data for cylinder cross-sectional area as well as height improves the correlation with body composition outcomes, as shown previously for body fatness in adults [18]. Nonetheless, this approach still could not match the accuracy of conventional BIA for predicting FFM, and it was only by combining the two approaches that the prediction of both tissue masses improved.

A combined approach to BIA would be easy to operationalize without greatly complicating the quick and simple protocol for data collection, even in young patients, which is a key strength of BIA as a technique. Simply by adding in the 3 additional girths, both BIVA_conventional_ and BIVA_specific_ parameters could be obtained, and the same data also allow other analytical approaches to be used, including the assessment of phase angle as a proxy for cellular health [29], and Piccoli's graphical approach which provides information on hydration status [13,33].

The strengths of our study include the high-quality measurements of body composition obtained using the four-component model, the relatively large sample size, and the wide range of body composition and BMI assessed. Restricting the analysis to children of European ancestry avoided the potential complication of ethnic variability in shape.

However, there are also some limitations to our analysis. At this stage, we do not know how much ethnicity might influence BIVA_specific_, as has already been assessed for conventional BIA in the pediatric age range [[6], [7], [8]]. Second, we have not considered children aged under 5 years, though this population has particular need of simpler protocols. Third, we have not yet addressed patients, in whom the need for simpler protocols is again particularly important. Moreover, we do not know how perturbations of body composition associated with illness (dehydration, wasting, oedema) might affect the ability of BIVA_specific_ parameters to index body fatness. Further work in younger children and patients is therefore required to fully appreciate the potential of this BIA variant for clinical assessment.

In summary, our analysis shows for the first time that conventional BIA and the new BIVA_specific_ approach make independent and additive contributions to predicting body composition variability in healthy children and adolescents across a wide range of age and BMI. Further work may extend this approach to patients, and may potentially improve the accuracy of BIA for predicting body composition variability in those most in need of such measurements.

## CRediT authorship contribution statement

The body composition studies that provided data were conceived and conducted by JCW, MF and JEW. JW conceived the analysis plan, derived the specific BIVA variables and ran the statistical analyses. All authors discussed the analyses and contributed to writing the manuscript.

## Funding

This research was supported by the NIHR Great Ormond Street Hospital Biomedical Research Centre, and received funding from the UK Medical Research Council.

## Data availability

The dataset analysed for the current study is available as a supplementary excel file.

## Conflict of interest

The Quadscan BIA instrument used in this study was received gratis from Bodystat. The manufacturer had no input into the design, conduct or analysis of the study. Other authors declare no conflicts of interest.
